# Biosensor-aided high-throughput screening of hyper-producing cells for malonyl-CoA-derived products

**DOI:** 10.1186/s12934-017-0794-6

**Published:** 2017-11-02

**Authors:** Heng Li, Wei Chen, Ruinan Jin, Jian-Ming Jin, Shuang-Yan Tang

**Affiliations:** 1 CAS Key Laboratory of Microbial Physiological and Metabolic Engineering, State Key Laboratory of Microbial Resources, Institute of Microbiology, Chinese Academy of Sciences, Beijing, China; 2 Beijing Key Laboratory of Plant Resources Research and Development, Beijing Technology and Business University, Beijing, China; 3 University of Chinese Academy of Sciences, Beijing, China

**Keywords:** Whole-cell biosensor, Malonyl-CoA, High-throughput screening, Triacetic acid lactone, Phloroglucinol

## Abstract

**Background:**

Malonyl-coenzyme A (CoA) is an important biosynthetic precursor in vivo. Although *Escherichia coli* is a useful organism for biosynthetic applications, its malonyl-CoA level is too low.

**Results:**

To identify strains with the best potential for enhanced malonyl-CoA production, we developed a whole-cell biosensor for rapidly reporting intracellular malonyl-CoA concentrations. The biosensor was successfully applied as a high-throughput screening tool for identifying targets at a genome-wide scale that could be critical for improving the malonyl-CoA biosynthesis in vivo. The mutant strains selected synthesized significantly higher titers of the type III polyketide triacetic acid lactone (TAL), phloroglucinol, and free fatty acids compared to the wild-type strain, using malonyl-CoA as a precursor.

**Conclusion:**

These results validated this novel whole-cell biosensor as a rapid and sensitive malonyl-CoA high-throughput screening tool. Further analysis of the mutant strains showed that the iron ion concentration is closely related to the intracellular malonyl-CoA biosynthesis.

**Electronic supplementary material:**

The online version of this article (10.1186/s12934-017-0794-6) contains supplementary material, which is available to authorized users.

## Background

Malonyl-coenzyme (CoA) is an essential building block for the biosynthesis of natural products, including fatty acids, polyketides, stilbenes and flavonoids, by providing the two-carbon units. Many of these compounds show beneficial properties for medical applications, including anti-cancer, anti-bacterial, antiviral, anti-inflammatory and anti-allergic activities, as well as in the development of agricultural products (e.g., insecticides) [1–5]. Several recent studies have reported the biosynthesis of these compounds in *Escherichia coli* [6–8]. Indeed, *E. coli* has become a widely used host for the industrial production of chemicals and fuels given that its genetic background has been extensively studied and molecular manipulation techniques with this bacterium are highly developed. However, since the intracellular malonyl-CoA level in *E. coli* is only around 0.07 nmol/mg dry cell weight [9], enhancement is needed for it to be effectively employed in specific biosynthesis applications.

**Fig. 1:**
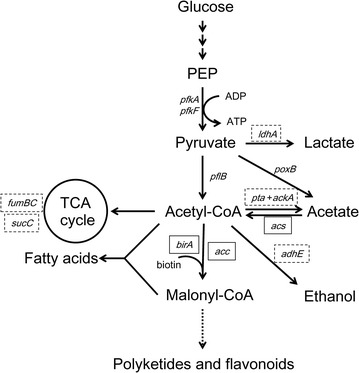
Malonyl-CoA metabolization. Genes whose inactivation or overexpression was previously reported to lead to increased malonyl-CoA levels are shown in a dashed-line box or solid box, respectively. *PEP* phosphoenolpyruvate

To this end, metabolic engineering strategies to elevate the intracellular malonyl-CoA biosynthesis in *E. coli* have been reported (Fig. 1). The main carbon flow from pyruvate and acetyl-CoA flows into the tricarboxylic acid (TCA) cycle, and only a small portion transforms into malonyl-CoA which participates in fatty acid biosynthesis [10]. In addition, pyruvate and acetyl-CoA are shunted to lactate and acetate respectively. Acetyl-CoA carboxylase (ACC) is proposed to be a major rate-controlling enzyme for malonyl-CoA biosynthesis [11]. ACC catalyzes the first committed step of the fatty acid synthetic pathway, and its overexpression proved to be effective to increase both the intracellular level of malonyl-CoA and the rate of fatty acid synthesis [12]. Based on ACC overexpression, FadA and FadB were also overexpressed to further increase the content of free fatty acids [13]. In addition to ACC, Rathnasingh et al. overexpressed the biotinilase BirA to active AccB, a subunit of ACC, by providing biotin [14]. Moreover, the lactate and acetate branching pathways can be blocked by deletion of the lactate dehydrogenase (*ldhA*), phosphotransacetylase (*pta*) [15] and acetate kinase (*ackA*) genes. Zha et al. reported that deletion of a second competing pathway (*ΔadhE*) that produces byproduct ethanol from acetyl-CoA also led to a slightly greater increase of the cellular malonyl-CoA concentration [9]. They further proved that the dual manipulation of acetate production from acetyl-CoA, involving assimilation [acetyl-CoA synthetase (Acs) overexpression] and pathway knockout (*ΔptaΔackA*), showed a synergistic effect in combination with ACC overexpression, resulting in a 15-fold improvement in the intracellular level of malonyl-CoA compared with that detected in wild type *E. coli*. Although some genes playing an important role in mediating the intracellular malonyl-CoA level have been identified through rational design strategies, a high-throughput genome-wide screen for genes that affect the metabolic flux flowing to malonyl-CoA has not yet been attempted because of the lack of appropriate screening tools.

**Fig. 2:**
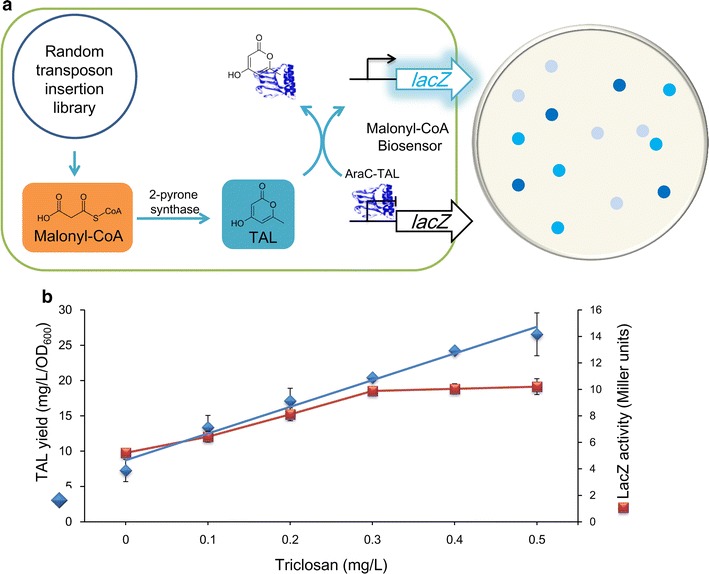
**a** Schematic diagram of the whole-cell biosensor of malonyl-CoA as a high-throughput screening tool. **b** Positive correlation between intracellular malonyl-CoA concentration (adjusted by triclosan) and TAL yield or LacZ activity (linear correlation coefficient = 0.9933 from 0 to 0.3 mg/L triclosan)

Malonyl-CoA is usually quantified by liquid chromatography–mass spectrometry (LC–MS) [16]. In the present study, we aimed to develop a high-throughput screening tool to facilitate a genome-wide screen of targets influencing the intracellular malonyl-CoA biosynthesis. In brief, a whole-cell biosensor for malonyl-CoA was developed based on a mutated AraC regulatory protein that is responsive to triacetic acid lactone (TAL) [17]. We then validated the whole-cell biosensor as a screening tool for rapidly and sensitively selecting *E. coli* strains showing hyper-production of malonyl-CoA from a random transposon insertion library (Fig. 2a). Use of this system is expected to provide novel gene targets related to the intracellular malonyl-CoA biosynthesis, and thus extend the biosynthetic applications of *E. coli*.

## Results

### Design and validation of a whole-cell biosensor of malonyl-CoA

To effectively monitor the malonyl-CoA biosynthesis in *E. coli*, a malonyl-CoA whole-cell biosensor was developed based on a mutated transcriptional regulatory protein AraC that is responsive to TAL (AraC-TAL) [17]. The mutated protein, AraC-TAL, was previously reported to regulate the expression of the *lacZ* gene controlled by the P_BAD_ promoter in a TAL concentration-dependent manner. Here, we used the heterologous expressed 2-pyrone synthase (2-PS) mutant S1 [17] to produce TAL using malonyl-CoA as a substrate. Therefore, the intracellular malonyl-CoA level was reflected by the TAL concentration, which can be rapidly reported according to the measurement of LacZ activity (Fig. 2a). Thus, the whole-cell biosensor of malonyl-CoA constructed by combining the AraC-TAL regulatory system and the 2-PS variant S1 can be used for in vivo high-throughput screening of malonyl-CoA.

To evaluate the validity of the high-throughput screening method, we treated the *E. coli* strain with triclosan, which is a broad-spectrum antibacterial and antifungal agent that blocks lipid synthesis in *E. coli* [18]. Specifically, triclosan inhibits the incorporation of malonyl-CoA into the cellular free fatty acid synthesis by inhibiting *Fab*I activity [19]. Due to the reduction in fatty acid synthesis, its precursor malonyl-CoA could accumulate. Thus, strains treated with different concentrations of triclosan were expected to exhibit different intracellular malonyl-CoA biosynthetic capabilities, resulting in different TAL production levels (Fig. 2b). As expected, in the triclosan concentration range of 0.1–0.5 mg/L, the TAL titers increased from 7.3 to 26.6 mg/L (Fig. 2b). There was also a good linear correlation detected between the triclosan concentration and the LacZ activity under 0–0.3 mg/L triclosan, confirming that the LacZ activity can be used to report the intracellular malonyl-CoA biosynthesis in this range (Fig. 2b).

### Genome-wide screening for targets related to the intracellular malonyl-CoA biosynthesis

To rapidly identify the target genes whose inactivation could improve the intracellular malonyl-CoA biosynthesis at a whole-genome scale, a transposon insertion library was constructed in strain BW-WT (with wild-type *araC* deleted and the P_BAD_-*lacZ* construct integrated). The plasmid pS1 expressing the 2-PS variant S1 and AraC-TAL was then used to transform the mutagenesis library. Certain gene disruptions influencing the malonyl-CoA biosynthesis could be reflected by measurement of the LacZ activity. Thus, mutant strains with a high intracellular malonyl-CoA biosynthetic capability could be obtained by simply selecting the bluest clones on LB agar supplemented with 5-bromo-4-chloro-3-indolyl β-d-galactopyranoside (X-GAL). Since the LacZ activity will saturate under relatively high malonyl-CoA concentrations (Fig. 2b), the mutant strains with high efficiency for malonyl-CoA synthesis could be selected at an early stage of cell growth on LB agar.

**Fig. 3:**
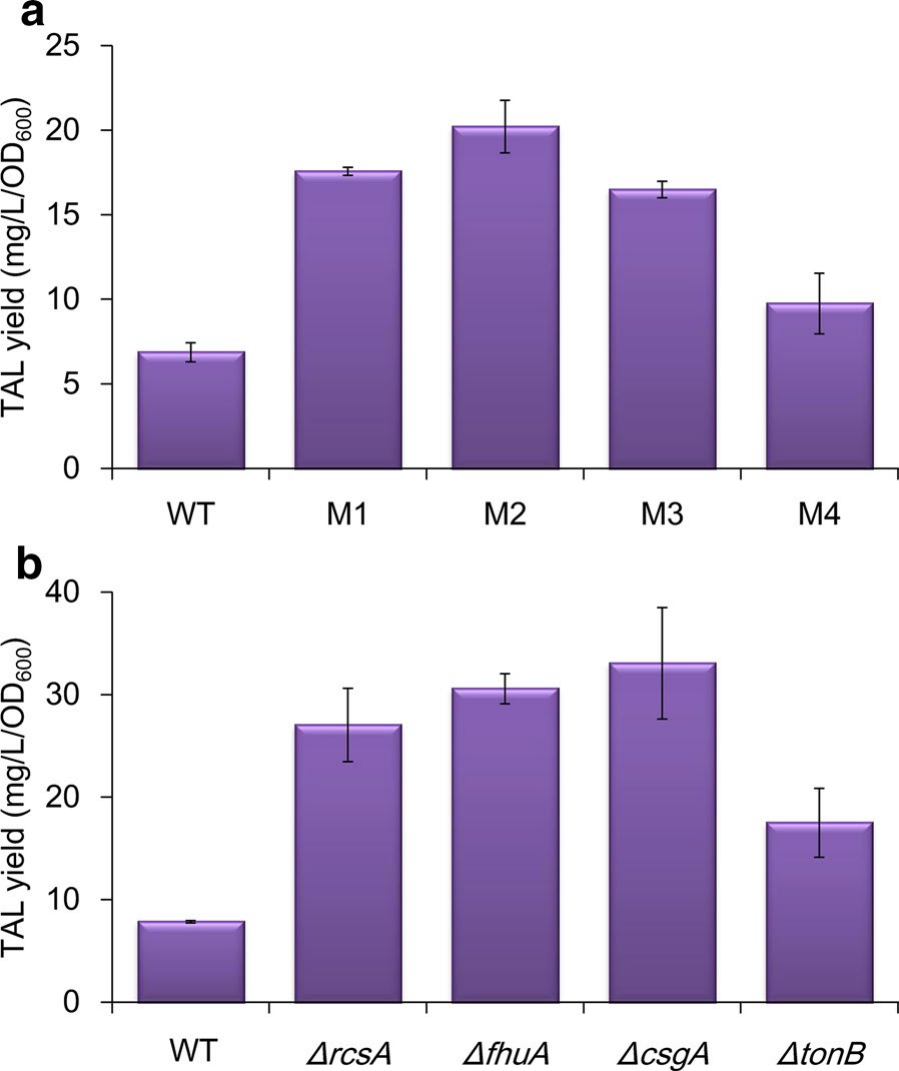
**a** TAL yields of mutant strains selected from the random transposon insertion library. P < 0.01. **b** TAL yields of mutant strains with the indicated genes deleted. P < 0.05

After culturing the transposon insertion library containing ~ 2 × 10^5^ mutant strains on LB agar supplemented with X-GAL for 18 h, four of the darkest blue clones (detected by naked eye) were selected and re-screened in liquid cultures to quantify TAL production (Additional file 1: Figure S1). These clones produced 1.4–3.0-fold more TAL than those produced by strain BW-WT (Fig. 3a). The locations of the transposon insertions in the selected mutant strains were identified. The four target genes inactivated by the transposons, *rcsA*, *fhuA*, *csgA* and *tonB* were individually knocked out in strain BW25113, resulting in strains *ΔrcsA*, *ΔfhuA*, *ΔcsgA*, and *ΔtonB*, respectively, and the enhanced TAL formation was confirmed in the deletion mutant strains (Fig. 3b). The TAL production levels in the deletion strains were 2.2–4.2-fold higher than that in the wild-type BW25113 strain.

### Iron ion concentration influences the intracellular malonyl-CoA biosynthesis

FhuA and TonB are both involved in the formation of the ferrichrome outer membrane transport complex. FhuA is an outer membrane porin that plays a role in the transport of ferrichrome across the outer membrane [20]. Conformational changes in the outer exposed-surface loops of FhuA brought about by TonB then promote the movement of siderophore-iron complexes across the outer membrane into the periplasm [21]. Thus, FruA and TonB both take part in the absorption of iron in *E. coli*. Accordingly, disruptions of the *fhuA* and *tonB* genes could improve the intracellular malonyl-CoA biosynthesis, which implies that iron concentration has an influence on the intracellular malonyl-CoA biosynthesis.

**Fig. 4:**
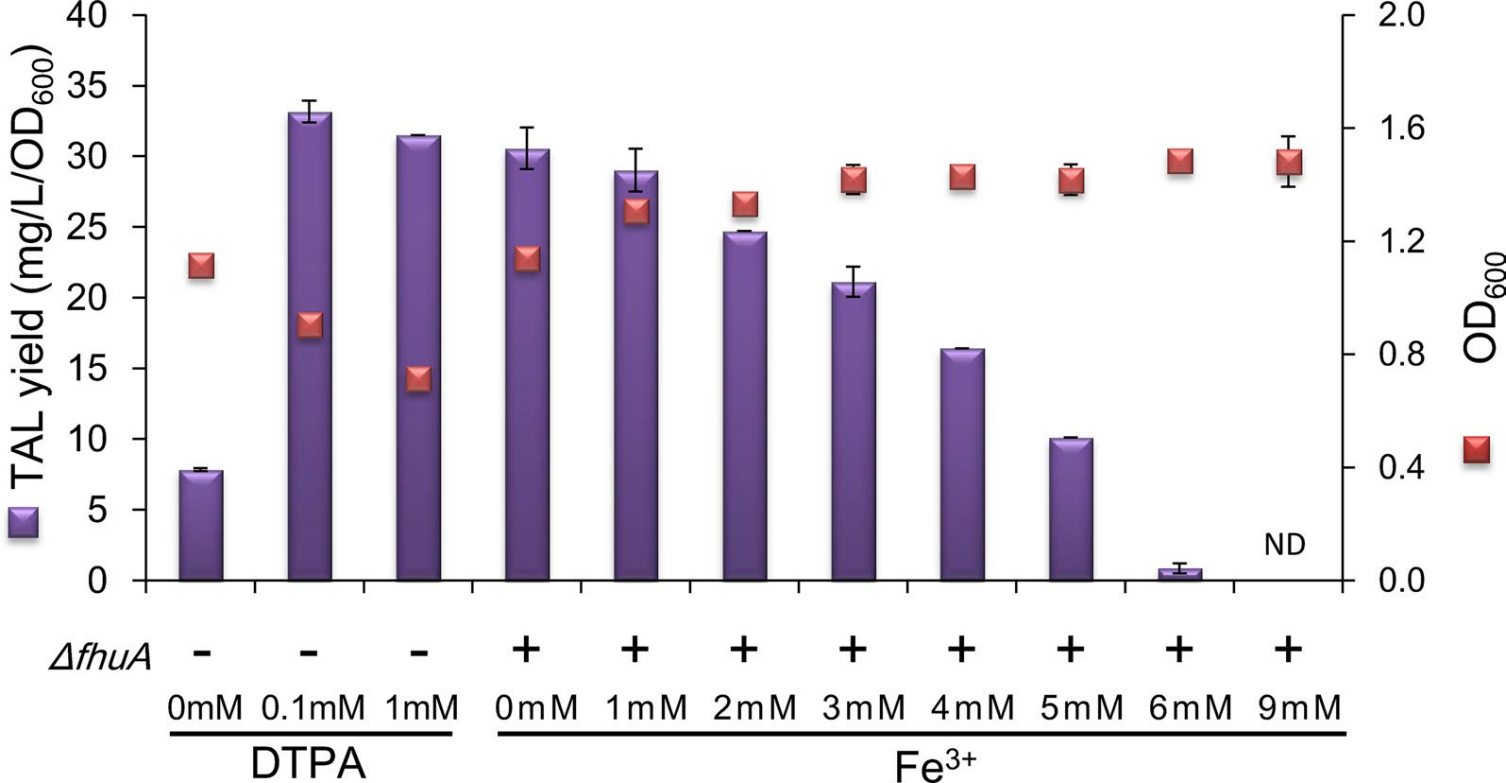
TAL yields and cell growth of the wild-type strain treated with DTPA and the *ΔfhuA* strain treated with iron ions. P < 0.01

To further explore the relationship between the intracellular malonyl-CoA biosynthesis and iron concentration, an iron chelator, diethylene triaminepentaacetic acid (DTPA), was used to sequester the extracellular iron of the wild-type BW25113 strain [22]. As expected, iron starvation promoted the TAL formation to a level exceeding that produced by the *ΔfhuA* strain, although cell growth was inhibited with DTPA treatment (Fig. 4). When the iron ion was supplemented in the medium of strain *ΔfhuA*, TAL production was reduced with increasing iron concentrations, and the production level was almost non-detectable in the presence of 9 mM of iron ions (Fig. 4).

### Production of phloroglucinol and fatty acids in the mutant strains

**Fig. 5:**
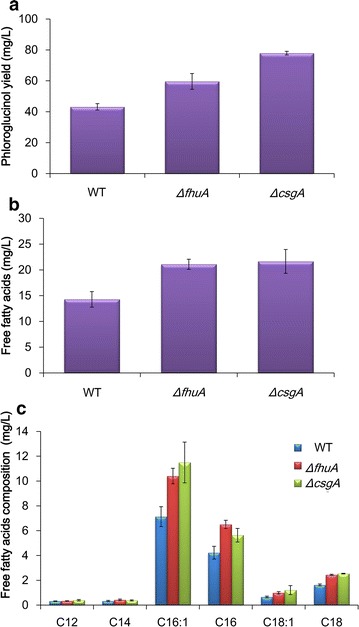
Yields of phloroglucinol (**a**) and free fatty acids (**b**) from the wild-type and mutant strains. **c** The compositions of free fatty acids. P < 0.05

To confirm the elevated intracellular malonyl-CoA biosynthetic capabilities in the selected mutant strains, another type III polyketide synthase from *Pseudomonas fluorescens*, phloroglucinol synthetase PhlD, was introduced into the *ΔfhuA* and *ΔcsgA* strains. PhlD catalyzes the biosynthesis of phloroglucinol via condensation of three molecules of malonyl-CoA [23]. As shown in Fig. 5a, the phloroglucinol yields in the *ΔfhuA* and *ΔcsgA* strains increased by 40 and 80%, respectively, compared with that in the wild-type strain. Given that malonyl-CoA is the sole precursor for phloroglucinol synthesis, the improved phloroglucinol production in these mutant strains confirmed their elevated intracellular malonyl-CoA biosynthesis.

**Fig. 6:**
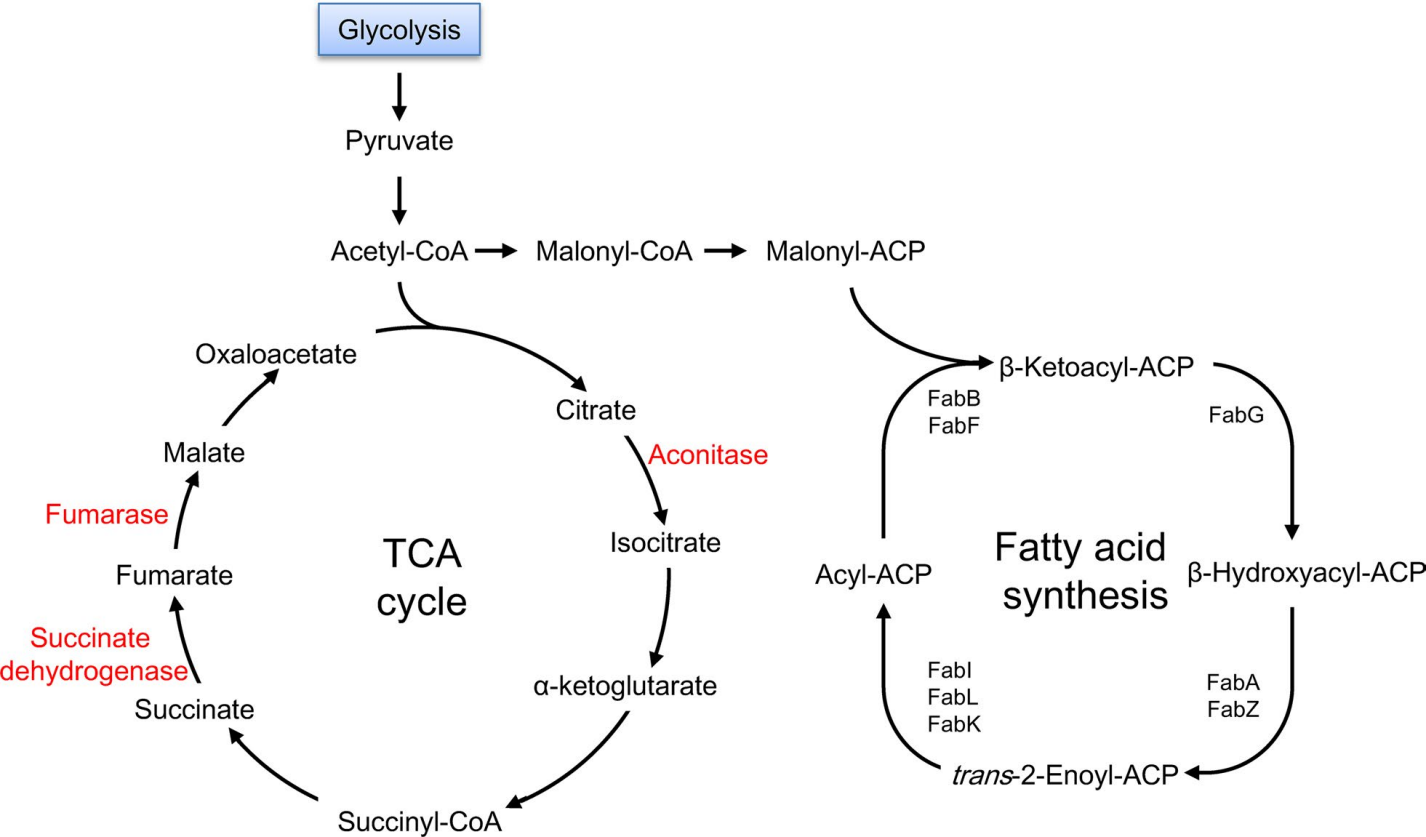
TCA cycle and fatty acid biosynthetic pathway. The reactions catalyzed by iron-sulfur (Fe/S) enzymes are indicated in red

Since malonyl-CoA is also the precursor of fatty acid biosynthesis in vivo, we determined the total free fatty acids contents in the mutant strains (Fig. 6). The results showed that the total free fatty acid levels in the *ΔfhuA* and *ΔcsgA* strains increased by 47 and 51%, respectively, as compared with that in wild-type BW25113 (Fig. 5b). The free fatty acid compositions are shown in Fig. 5c. C16:1 and C16 straight chain fatty acids were the main free fatty acid components identified, which accounted for ~ 80% of the total free fatty acids. The distribution of the free fatty acids composition of the mutant strains *ΔfhuA* and *ΔcsgA* displayed a similar pattern to that of the wild-type strain.

## Discussion

A whole-cell biosensor of malonyl-CoA was developed in this study, making use of the TAL-responsive AraC regulatory protein mutant AraC-TAL. Using the biosensor as a high-throughput screening tool, mutant strains with improved malonyl-CoA biosynthetic capabilities were successfully selected from a transposon insertion mutagenesis library. Further investigation of the selected beneficial strains demonstrated that iron concentration is critical for the intracellular malonyl-CoA biosynthesis, which is a novel finding of this study. It is possible that the TCA cycle becomes inhibited in the presence of an extremely low level of iron given that several enzymes in this cycle are iron-sulfur (Fe/S) proteins [24–28], which directed more acetyl-CoA to form malonyl-CoA (Fig. 6).

RcsA is a positive activator of colanic acid capsular polysaccharide synthesis, and interacts with RcsB to optimize the transcription of genes involved in the synthesis of colanic acids [29]. Therefore, it is possible that the inactivation of RcsA would weaken the biosynthesis of colanic acids, thus leading to a higher flux of glycolysis to in turn influence the malonyl-CoA biosynthesis. CsgA is the major curli subunit. However, the effect of *csgA* deletion on intracellular malonyl-CoA biosynthesis is not yet clear.

A regulatory protein directly responsive to malonyl-CoA, FapR, has been previously reported, which also shows potential to be used to report the intracellular malonyl-CoA concentration [30, 31], or to dynamically control the biosynthetic pathways of malonyl-CoA-derived products [32–35]. However, although the intracellular pool of malonyl-CoA may vary to some extent, as a metabolic intermediate, endogenously produced malonyl-CoA would be metabolized by metabolic pathways and would not accumulate to very high levels. Thus, FapR, which directly senses the intracellular malonyl-CoA concentration, may not be appropriate to report the total capability of a cell for synthesizing malonyl-CoA. Using the novel biosensor developed in this study, malonyl-CoA was converted to a stable and non-metabolized product, TAL, whose concentration can be used to estimate the amount of malonyl-CoA produced by the cell during a certain period of time. Therefore, this biosensor is expected to more effectively select strains capable of synthesizing high amounts of malonyl-CoA for the biosynthesis of related natural products.

## Conclusion

A whole-cell biosensor was developed to rapidly report the intracellular malonyl-CoA level. The biosensor was successfully applied to screen a transposon insertion library of strain BW-WT and could identify targets related to the intracellular malonyl-CoA biosynthesis at a genome-wide scale. These selected mutant strains were verified to produce higher levels of free fatty acids, as well as higher titers of TAL or phloroglucinol when the corresponding synthase gene was introduced. Collectively, these results confirmed that the mutant strains exhibit higher intracellular malonyl-CoA biosynthetic capabilities and are applicable for natural product biosynthesis using malonyl-CoA as precursor.

## Methods

### General

DNA polymerase, T4 DNA ligase, and restriction endonucleases were purchased from Takara Bio Inc (Dalian, China). Oligonucleotides were synthesized by Life Technologies (Shanghai, China). TAL and triclosan was purchased from Sigma-Aldrich (St. Louis, USA).

### Plasmid construction

The strains and plasmids used in this study are presented in Table 1. The primers used for the plasmid construction are listed in Table 2. Plasmid pTn10 was constructed as follows. Fragment F1 containing genes tnp* encoding Tn10 transposase (Genbank Accession No. AF310136) under the control of P_*tac*_ promoter, and *mob* gene encoding Mob protein (Genbank Accession No. NZ_LSAZ01000024) was synthesized by GENEWIZ (Suzhou, China). After digestion with *Xba*I and *Xho*I, fragment F1 was ligated into the PCR product amplified with primers GEX-*Xho*I-fwd and GEX-*Xba*I-rev using plasmid pGEX-6P-1 (GE Healthcare) as template, resulting in plasmid pGEX-TM. Fragment F2 containing R6K replication origin was amplified with primers R6K-*Not*I-fwd and R6K-overlap-rev using plasmid pAH156 [36] (Genbank Accession No. AY048737) as template. Fragment F3 containing IS10L, gene *kan*, and IS10R [37] was amplified using plasmid pSM04 [38] as template with primers IS-overlap-fwd and IS-*Sac*II-rev. Fragment F2 and F3 were PCR-assembled with primers R6K-*Not*I-fwd and IS-*Sac*II-rev, resulting in fragment F4. After digestion with *Not*I and *Sac*II, fragment F4 was ligated into the PCR product amplified with primers *lac*I-*Sac*II-fwd and amp-*Not*I-rev using plasmid pGEX-TM as template, resulting in plasmid pTn10. The gene encoding AraC-TAL (P8 V, T24I, H80G, Y82L, H93R) [17] was synthesized by GENEWIZ (Beijing, China). After digested with *Nde*I and *Xho*I, it was subsequently ligated into plasmid pSM02 [38], resulting in plasmid pSM02M. The mutated *g2ps1* gene encoding the 2-PS variant S1 [17] was synthesized by GENEWIZ. It was digested with *Xho*I and *Eco*RI and subsequently ligated into plasmid pSM02M, resulting in plasmid pS1. The *phlD* gene (Genbank Accession No. EU554263.1) was synthesized by GENEWIZ (Beijing, China). The gene was digested with *Nco*I and *Sac*I and subsequently ligated into plasmid pSM01, resulting in plasmid pPhlD.

**Table 1:** Plasmids and strains used in this study

Strains and plasmids	Description	Source
Plasmids		
pS1	Genes encoding AraC-TAL and 2-PS variant S1 under the control of promoter P_*tac*_. Amp^r^	This study
pTn10	Transposon Tn10, Kan^r^	This study
pPhlD	Gene *phlD* under the control of P_BAD_, Kan^r^	This study
Strains		
BW-WT	BW25113 (*ΔaraC*), with promoter P_BAD_ controlled *lacZ* integrated	This study
*ΔrcsA*	BW25113 (*ΔrcsA*)	This study
*ΔfhuA*	BW25113 (*ΔfhuA*)	This study
*ΔcsgA*	BW25113 (*ΔcsgA*)	This study
*ΔtonB*	BW25113 (*ΔtonB*)	This study

**Table 2:** Primers used in this study

Primer name	Sequences (5′-3′)
GEX-*Xho*I-fwd	CGGCTCGAGTGACGATCTGCCTCGCGCGT
GEX-*Xba*I-rev	TGCTCTAGATCACTGCCCGCTTTCCAGTC
R6K-*Not*I-fwd	TAAGCGGCCGCCTAATTCCCATGTCAGCCGT
R6K-overlap-rev	GACAAGATGTGTATCCACCTTAACTAAGATCCGGCCACGATGCGTCCGGC
IS-overlap-fwd	ATCGTGGCCGGATCTTAGTTAAGGTGGATACACATCTTGTCATATGATCTACTAGAGCTGATCCTTCAAC
IS-*Sac*II-rev	AAACCGCGGAGTTAAGGTGGATACACATCTTGTCATATGATCTTAGAAAAACTCATCGAGCA
lacI-*Sac*II-fwd	AAACCGCGGTATTTTCTCCTTACGCATCT
*amp*-*Not*I-rev	TAGGCGGCCGCTTACCAATGCTTAATCAGTG
*lacZ*-F	TTTAAGAAGGAGATATACATATGACCATGATTACGGATTC
*lacZ*-R	TTATTTTTGACACCAGACCA
P_BAD_-F	CCATAAGATTAGCGGATCCT
P_BAD_-R	GAATCCGTAATCATGGTCATATGTATATCTCCTTCTTAAA
pAH156-P_BAD_-F	CTCTAGATAAGGAGGAAAAACTCGAGCCATAAGATTAGCGG
pAH156-*lacZ*-R	CTCGGTACCCGGGGATCCGCTCTCGAGGTCGACGGTATCG
*lacZ*-pAH156-F	CGATACCGTCGACCTCGAGAGCGGATCCCCGGGTACCGAG
P_BAD_-pAH156-R	CCGCTAATCTTATGGCTCGAGTTTTTCCTCCTTATCTAGAG
kan-sp1	TATCAGGACATAGCGTTGGCTACCCG
kan-sp2	CGGCGAATGGGCTGACCGCTTC
kan-sp3	GTGCTTTACGGTATCGCCGCTC
sp-seq	CATCGCCTTCTATCGCCTTCTT
AD1	NGTCGASWGANAWGAA
AD2	TGWGNAGSANCASAGA
AD3	AGWGNAGWANCAWAGG
AD4	STTGNTASTNCTNTGC

Restriction sites are underlined

N, A or C or G or T; S, G or C; M, A or C; W, A or T

### Strain construction

Strains used in this study are listed in Table 1. Gene *lacZ* was amplified using the genomic DNA of strain MG1655 as template with primers *lacZ*-F and *lacZ*-R. Promoter P_BAD_ was amplified with primers P_BAD_-F and P_BAD_-R using plasmid pSM04 [39] as template, The two PCR products were overlapped, and amplified again with primers pAH156-P_BAD_-F and pAH156-*lacZ*-R, resulting in fragment P_BAD_-*lacZ*. PCR was performed with primers *lacZ*-pAH156-F and P_BAD_-pAH156-R using the CRIM plasmid pAH156 [36] as template. The PCR product was assembled with fragment P_BAD_-*lacZ* by Gibson Assembly [39]. The construct was then integrated into the chromosome of strain BW1A (strain BW25113 with gene *araC* deleted) [38], using helper plasmid pAH69 [36], resulting in strain BW-WT. The integration was verified by PCR. Deletion of *E. coli* chromosomal genes, *rcsA*, *fhuA*, *csgA* and *tonB* was carried out via P1 phage transduction using phage libraries of strain JW1935-1, JW0146-2, JW1025-1 and JW5195-1 (Keio collection) [40], and then the FRT-flanked *kan* gene was removed using flipase-mediated recombination [41], resulting in strains *ΔrcsA*, *ΔfhuA*, *ΔcsgA* and *ΔtonB*, respectively.

### Construction and screening of random transposon insertion library

Strain BW-WT harboring plasmid pS1 carrying genes encoding AraC-TAL and the 2-PS variant S1 was transformed with plasmid pTn10, and then plated on LB agar containing 1 mM IPTG. After incubation at 37 °C for 16 h, the darkest blue colonies (by the eye) were selected and cultured in 3 mL LB for LacZ activity assay and TAL quantification using HPLC.

To identify the location of the transposon insertion in the chromosome, the thermal asymetric interlaced (TAIL)-PCR was conducted as described [42, 43]. Strains exhibiting increased shikimic acid productions were cultured and harvested to isolate genomic DNA. 100 ng of the genomic DNA was used as template for TAIL-PCR. Genomic sequences flanking the transposon were amplified with a mixture of four arbitrary degenerate (AD) primers (AD1-4), plus the specific primer inside the *kan* gene, kan-SP1, kan-SP2 and kan-SP3, individually. Three rounds of TAIL-PCR cycling were performed. The tertiary TAIL-PCR product was purified and sequenced using primer sp-seq. The sequence obtained was blasted in the database of *E. coli* K-12 strain W3110 (http://ecocyc.org/) to identify the location of the transposon insertion in the chromosome. The location was further verified using PCR with primers kan-SP3 plus the primer designed according to the chromosome DNA sequence close to the insertion point.

### β-Galactosidase activity assay

β-Galactosidase activity was assayed as described by Miller [44], with some modifications. The reaction mixture was prepared by addition of 650 μL buffer Z, 40 μL of 12 mM 2-nitrophenyl-β-d-galactopyranoside (*o*NPG) and 10 μL of the cell lysate. After incubation at 37 °C for 10 min, the reaction was terminated by the addition of 185 μL of 1 M sodium carbonate. The absorbance at 420 nm was measured with a SynergyMx Multi-Mode Microplate Reader (BioTek, Vermont, USA). β-Galactosidase activity in Miller units was calculated as (1000 × OD_420_)/(T × V × OD_600_), where T represents the reaction time (minute) and V represents the volume of cell lysate used in the assay (mL).

### HPLC quantification

The concentration of TAL and phloroglucinol in the cell-free supernatant were measured by HPLC using a Shimadzu LC-20A system equipped with a photodiode array detector (Shimadzu Corp., Kyoto, Japan) operating at 280 nm (for TAL) or 265 nm (for phluroglucinol). Separation was achieved using a Waters Symmetry C18 column (250 × 4.6 mm, 5 µm) (Waters, USA) working at 30 °C with the mobile phase of 20% acetonitrile (containing 0.1% acetic acid) at a flow rate of 0.6 mL/min (for TAL) and the mobile phase of 10% methanol at a flow rate of 0.6 mL/min (for phloroglucinol).

### GC–MS identification of free fatty acids

Fatty acids were extracted from 400 µL of acidified culture with ethyl acetate and esterified with ethanol. Fatty ethyl esters were extracted with hexane and run on an HP 5890 chromatograph with FID detector using a DB-5 capillary column (length, 30 m; inner diameter, 0.25 mm; film thickness, 0.25 µm; J&W Scientific, Folsom, USA). The temperature program ran from 170 to 300 °C with an initial and final holding time of 2 min and a rate of 10 °C/min. The injector and detector temperatures were 240 and 300 °C, respectively. Helium was the carrier gas, with a linear flow velocity of 40 mL/min at 100 °C.

## Additional file

**Additional file 1: Figure S1.** Dark blue clones selected from the transposon insertion library grown on LB agar supplemented with X-GAL, together with the wild-type strain.

## Abbreviations

ACC: acetyl-CoA carboxylase
DTPA: diethylene triaminepentaacetic acid
TAL: triacetic acid lactone
TCA: tricarboxylic acid
X-GAL: 5-bromo-4-chloro-3-indolyl β-d **-**galactopyranoside
