# Supplementary figures and images for: Biosensor-aided high-throughput screening of hyper-producing cells for malonyl-CoA-derived products
Source: Microb Cell Fact. 2017 Nov 2;16:187. doi: 10.1186/s12934-017-0794-6 (PMC5669015; doi:10.1186/s12934-017-0794-6)

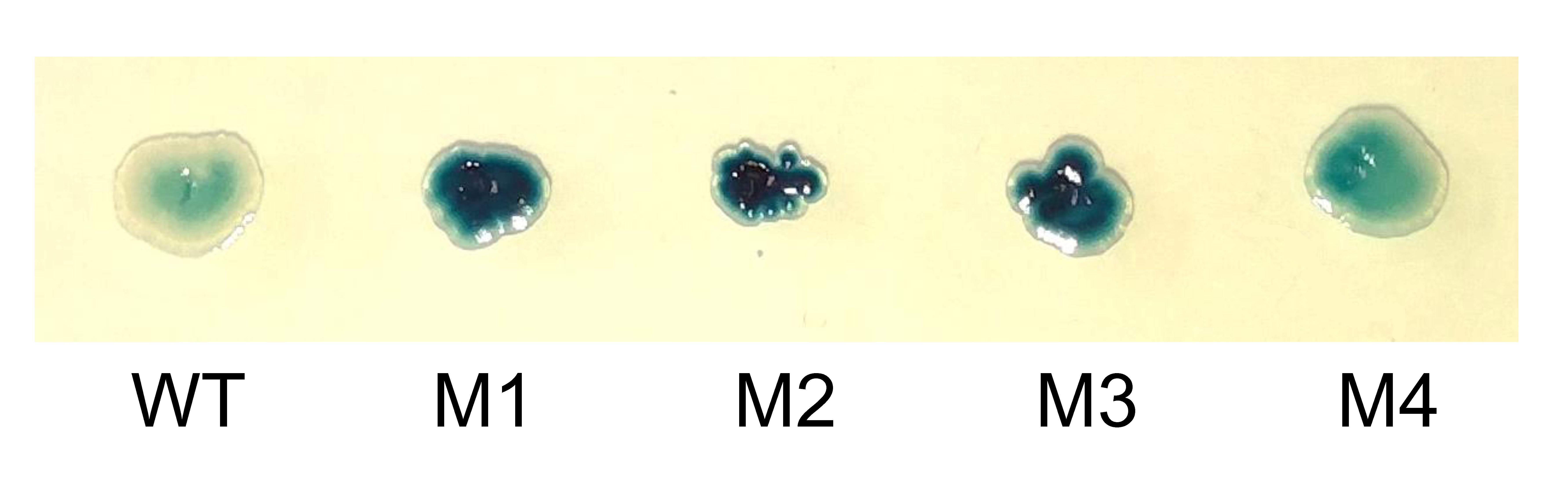

Supplement: Supplementary file 1 — Additional file 1: Figure S1. Dark blue clones selected from the transposon insertion library grown on LB agar supplemented with X-GAL, together with the wild-type strain. [file 12934_2017_794_MOESM1_ESM.tif]
